# Electrochemical performances of MnO_2_/Fe_3_O_4_/activated carbon ternary composites for supercapacitor and direct ethanol fuel cell catalyst application†

**DOI:** 10.1039/d5ra02075a

**Published:** 2025-05-16

**Authors:** Yilkal Dessie, Eneyew Tilahun Bekele, Bedasa Abdisa Gonfa, C. R. Ravikumar, Syed Khasim, Getahun Abraham

**Affiliations:** a Department of Applied Chemistry, College of Applied Natural Science, Adama Science and Technology University 1888 Adama Ethiopia yilikaldessie@gmail.com; b Research Centre, Department of Science, East-West Institute of Technology Bangalore 560091 India; c Advanced Materials Research Laboratory, Department of Physics, Faculty of science, University of Tabuk Tabuk 71491 Saudi Arabia; d Department of Chemistry, College of Natural and Computational Sciences, Kebri Dehar University P. O. Box 250 Kebri Dehar Ethiopia

## Abstract

MnO_2_ and Fe_3_O_4_ nanoparticles' slow ion-diffusion kinetics and weak electrical conductivity hinder their electrochemical performance in supercapacitors and their energy-conversion ability in direct ethanol fuel cells (DAFCs). Combining MnO_2_/Fe_3_O_4_ with biomass-based activated carbon (AC), which is conductive, inexpensive, and has a long cycle life, to create MnO_2_/Fe_3_O_4_/AC can improve their electrochemical performances for ethanol oxidation and in supercapacitors. Herein, MnO_2_/Fe_3_O_4_/AC ternary composites were synthesized *via* a facile method. The physicochemical and electrochemical properties of pure and composite materials were characterized using TGA-DTA, XRD, FTIR, BET, SEM-EDX, TEM, HRTEM, SAED, CV, and EIS. The composite MnO_2_/Fe_3_O_4_@8%AC exhibited the highest specific capacitance, with a value of 515.113 F g^−1^ at 1 A g^−1^. Furthermore, in cyclability tests, after 700 cycles at a current density of 1 A g^−1^, its charge-storage performance showed an 81.83% capacity retention and a maximum energy density of 27.679 W h kg^−1^. Upon integration in a DAFC, MnO_2_/Fe_3_O_4_@8%AC electrocatalytic ethanol oxidation was achieved with a maximum power density of 44.41 mW cm^−2^, indicating better performance than that of other pure and composite catalysts. Therefore, this work provides a potential candidate for use in efficient energy-storage devices and an effective catalyst electrode for the ethanol oxidation reaction.

## Abstract 2

MnO_2_/Fe_3_O_4_/AC ternary composites were synthesised and investigated for their physiochemical and electrochemical properties for potential applications in energy storage and conversion devices.

## Graphical abstract

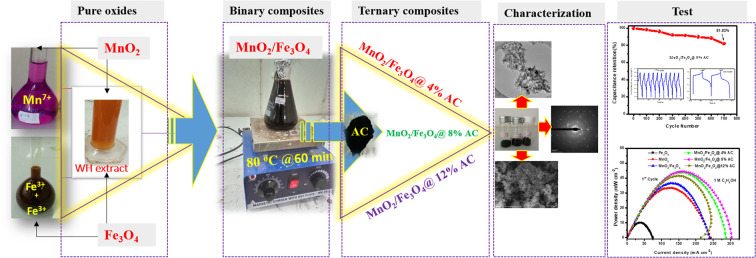

## 1 Introduction

In order to solve the issues brought on by the energy crisis and develop substitutes for traditional fossil fuels, sustainable energy systems are crucial for future strategic and economic progress.^1^ Among the renewable energy devices, supercapacitors^2^ and direct alcohol fuel cells (DAFCs)^3^ are regarded as promising energy-conversion and -storage devices. Concentrated efforts have been made to design and develop new innovative materials for energy storage because of the necessity for high energy and high power density. Supercapacitors, comprising electrochemical double-layer capacitors (EDLCs) and pseudocapacitors, have been receiving a lot of interest owing to their superior power densities, great cycling stability, and quick charging and discharging.^4^

However, storing charge in supercapacitors using low-cost and environmentally friendly nanomaterials with superior energy density is a major challenge at present. To solve this stated problem, the low cost, large potential window, environmental friendliness, and natural availability with high theoretical capacity of both MnO_2_ (1370 F g^−1^)^5^ and Fe_3_O_4_, including the presence of different valence states in magnetite (Fe_3_O_4_),^6^ nanoparticles make them among the most viable alternative electrode materials for high-performance supercapacitors. However, their poor electrical conductivity and slow ion-diffusion kinetics largely prevent their extensive practical use in supercapacitors. Recent studies have drawn the conclusion that the combination of MnO_2_ with Fe_3_O_4_ to form binary composites leads to their better stability and recyclability. Furthermore, surface functionalization further contributes to the stability and increases the specific surface area of Fe_3_O_4_ nanoparticles as it enables the formed binary composites with the synergy of supercapacitor.^7,8^ The poor in electrical conductivity and short life cycle in Fe_3_O_4_/MnO_2_ but having good energy density by storing the charge of the redox reaction; modification of Fe_3_O_4_/MnO_2_ binary composites with good conductive, low-cost, and long cycle life biomass-based activated carbon (AC) have been paid considerable attention for energy storage applications. Among the many carbonaceous materials, biomass-derived AC has a significant competitive advantage over supercapacitor electrodes due to its easy preparation, excellent capacitive features with high cyclic stability, tunable specific surface area (SSA), good conductivity, and hierarchical porous structure. Furthermore, biomass is considered an eco-friendly, sustainable, and renewable resource when used as a precursor to obtain activated carbon.^4^

One of the primary issues affecting the real-world implementation of DAFCs is the electroactivity of the anodic materials. Electrode materials with improved activity and durability for direct alcohol fuel cells, such as direct ethanol fuel cells (DEFCs), under basic or acidic conditions might be created using the low-cost catalytic activity and durability in the ethanol electro-oxidation processes.^3^ The expensive cost of metals, such as platinum (Pt), and palladium (Pd), restricts the common use of *e.g.* Pt electrodes as anodes in DEFCs, despite the fact that they exhibit effective catalytic activity for alcohol oxidation. It is reasonable to assume that the fuel cell kinetics would be enhanced and Pt-free electrocatalysts could be employed if the DEFCs could be run in an alkaline electrolyte rather than an acidic one. According to reports, Pd has greater activity than Pt in alkaline conditions and would be an effective electrocatalyst for the oxidation of ethanol.^9^ Consequently, many researchers have tried to study the electro-oxidation of ethanol by compositing Pt and Pd electrode materials.^10–14^

Considering the above-mentioned research studies, herein, we propose combining water hyacinth (WH) stem biomass-based AC^15^ with hybrid MnO_2_/Fe_3_O_4_ (1 : 1) binary composites to form a ternary composite structure with improved electrochemical performance for use in supercapacitors and ethanol oxidation in DEFCs through a remarkable physicochemical enhancement effect. Consequently, we constructed an efficient MnO_2_/Fe_3_O_4_ (1 : 1)@AC ternary composite electrode and systematically examined its electrochemical performances, including its specific capacitance and power density, with an aim to assess its potential for use in supercapacitors. In addition, we assessed the use of the MnO_2_/Fe_3_O_4_ (1 : 1)@AC ternary catalyst for the electro-oxidation of ethanol in an alkaline solution, with the aim that it could replace the current high-cost metals or alloys used in DEFCs by showing improved catalytic activities. The pure metal oxides (MnO_2_ (1 : 1) and Fe_3_O_4_ (1 : 1)), binary composite (MnO_2_/Fe_3_O_4_ (1 : 1)), and ternary composite (MnO_2_/Fe_3_O_4_ (1 : 1)@4% AC, MnO_2_/Fe_3_O_4_ (1 : 1)@8% AC, and MnO_2_/Fe_3_O_4_ (1 : 1)@12% AC)-modified catalysts all showed notable improvements in both supercapacitors and ethanol oxidation performance.

## 2 Experimental section

### 2.1. Chemicals

Analytical-grade reagents were employed as received during the solution preparations without any additional purification. Specifically, potassium permanganate (KMnO_4_, purity ≥99.9%), ferric chloride hexahydrate (FeCl_3_·6H_2_O, purity ≥98.5%), ferrous chloride hydrated (FeCl_2_·4H_2_O, purity ≥98%), potassium carbonate (K_2_CO_3_, purity ≥99%), poly(vinyl alcohol) (PVA, purity ≥87%), potassium hydroxide (KOH, purity ≥90%), sodium hydroxide (NaOH, purity ≥99%), hydrochloric acid (HCl) pure grade 37%, nickel foam (Ni foam, purity ≥98% porosity), absolute ethanol (C_2_H_5_OH, purity ≥99.5%), double distilled water, and water hyacinth (WH) stem as an activated carbon source were used.

### 2.2. Preparation of WH leaf extract

The WH leaf sample was collected from Koka Dam, East Shewa Zone, Ethiopia, and was prepared as follows.^16^ Briefly, the collected fresh leaf of WH was washed twice with distilled water to remove any surface impurities and dust, followed by air drying at room temperature in a shaded region. Then, 15 g of dried leaf was ground and heated in 400 mL of distilled water in a 1000 mL Erlenmeyer flask at 50 °C for 1 h. Finally, the extract was cooled and filtered using Whatman filter paper No. 1 and stored in a colored bottle in the refrigerator for later use.

### 2.3. Preparation of activated carbon from WH stem

The dried WH stem was cut into small pieces ≈2 cm and dried in sunlight for a week and then dried in an oven at 90 °C for 2 h.^17^ Following a previously reported activation procedure with little modification,^18^ the dried stem was crushed and pulverized to obtain the precursor samples with a particle size of 150 μm (mesh #100). Then, 15 g of the WH was treated using 1 : 2 (10 g K_2_CO_3_ + 20 g KOH), 1 : 1 (15 g K_2_CO_3_ + 25 g KOH), and 2 : 1 (20 g K_2_CO_3_ + 10 g KOH) mass ratios of K_2_CO_3_ to KOH. All ratios were kept in without air for about 24 h. Afterwards, the samples were washed with distilled water to adjust the pH to near neutral, and then dried in an oven at 90 °C for 2 h. To determine the stable thermal temperature for pyrolysis and carbonization, a 1 : 1 ratio of dried sample was tested using a thermogravimetric analysis (TGA) instrument. From the TGA results (see Fig. S1†), near 500 °C was identified as the highest stable temperature. Next, each dried sample was covered by aluminum foil and placed on a ceramic dish, which was then inserted in to a stainless tube, and pyrolyzed in the furnace at 500 °C for 1 h at a heating rate of 5 °C min^−1^. After the pyrolysis procedure, the furnace was cooled to room temperature. After pyrolysis, the samples were milled and sieved into smaller sizes in the range of 3–5 mm. Each of the smaller-sized activated carbon (AC) samples was subjected to acid treatment by mixing 1.0 g of carbon from each of the samples with different ratios with 100 mL of 2 M HCl solution. The aqueous solutions were stirred for about 1 h at 90 °C to remove as many inorganic impurities as possible. The samples were then rinsed until their pH was close to neutral and were then dried in an oven at 90 °C for 2 h. Finally, the prepared samples were denoted as WHAC (1 : 2), WHAC (1 : 1), and WHAC (2 : 1) for use in the further experiments.

### 2.4. Synthesis of MnO_2_ NPs

The MnO_2_ NPs were prepared by a green method following a previously reported procedure^19^ with a little modification after optimization. In brief, solutions were prepared in three ratios of 1 mM of KMnO_4_ to WH leaf extract (25 : 75, 50 : 50, and 75 : 25 v/v ratio). The resulting mixtures were stirred for about 70 min at 40 °C, with the pH of the suspension controlled at pH 7.15 by adding a 2 M solution of NaOH. Afterwards, the synthesized MnO_2_ NPs were separated three times by centrifugation at 3000 rpm for 30 min and washed with ethanol–distilled water–ethanol in sequence. The washed samples were then dried overnight in an oven at 40 °C. The 1 : 1 ratio dried sample was tested by TGA and 400 °C was selected as the calcination temperature. Finally, the three ratio samples, *i.e.*, MnO_2_ (1 : 3), MnO_2_ (1 : 1), and MnO_2_ (3 : 1) NPs, were calcined in a muffle furnace at 400 °C for 3 h. Among them, due to its smallest crystalline size (see Table S1†), MnO_2_ (1 : 1) was selected and used in the further experiments.

### 2.5. Synthesis of MnO_2_/Fe_3_O_4_ composites

MnO_2_/Fe_3_O_4_ composites were synthesized following a literature-reported procedure with little modification.^20^ First, to a solution containing 100 mL of 400 mM FeCl_3_·6H_2_O and 100 mL of 200 mM FeCl_2_·4H_2_O, 25 mL of WH extract was added with stirring for 2 h at 40 °C in a 1000 mL round-bottom flask, and the black precipitate formed was labelled as Sample A. Second, 200 mL of 400 mM KMnO_4_ solution was added to another 1000 mL round-bottom flask and 25 mL of WH extract was added with stirring for 2 h at 40 °C, and the formed brown precipitate was labelled as Sample B. Then, three different ratios (1 : 2, 1 : 1, and 2 : 1 v/v) of black (sample A) and brown (sample B) suspensions were mixed separately in 400 mL beakers. Subsequently, the resulting mixtures were continuously stirred and heated at 80 °C for approximately 60 min each in the water bath to initiate the reduction process. Thereafter, each of the suspensions was subjected to an additional 50 min blending with stirring but without any heat supply. After the 50 min, a 2 M solution of NaOH was added dropwise to attain pH 11, and the solutions were left under stirring for a further 15 min to regulate the solution's acidity, signifying the formation of a precipitate. The separation of this precipitate was accomplished using a centrifuge operating at 3000 rpm for 5 min. After obtaining the precipitate, it underwent several rinses with ethanol–distilled water–ethanol in sequence to eliminate any impurities. After drying in an oven at 40 °C, the 1 : 1 ratio sample was analyzed by TGA, and 450 °C was identified as the highest stable temperature. The three samples were then calcined at this temperature in a muffle furnace for 3 h. Finally, the samples were labelled as MnO_2_/Fe_3_O_4_ (1 : 2), MnO_2_/Fe_3_O_4_ (1 : 1), and MnO_2_/Fe_3_O_4_ (2 : 1). Among them, due to its smallest crystalline size (see Table S1†), MnO_2_/Fe_3_O_4_ (1 : 1) was selected and used for the further experiments.

### 2.6. Synthesis of MnO_2_/Fe_3_O_4_/AC composites

**Scheme 1:**
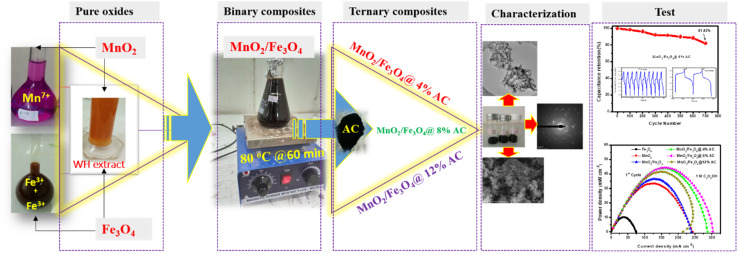
Schematic representation of the synthesis of pure and composite materials.

To prepare the ternary composites, the starting procedure was adopted from the process for preparing the MnO_2_/Fe_3_O_4_ binary composites with a little modification. Briefly, first, a solution containing 100 mL of 400 mM FeCl_3_·6H_2_O and 100 mL of 200 mM FeCl_2_·4H_2_O with 25 mL of WH extract was stirred for 2 h at 40 °C in a 1000 mL round-bottom flask, and the formed black precipitate was labelled as Sample A. Second, 200 mL of 400 mM KMnO_4_ solution was added to another 1000 mL round-bottom flask with 25 mL of WH extract and stirred for 2 h at 40 °C, and the formed brown precipitate was labelled as Sample B. Then, equal volumes (1 : 1 v/v) of the black (Sample A) and brown (Sample B) suspensions were mixed in a 400 mL beaker (*i.e.*, MnO_2_/Fe_3_O_4_ (1 : 1)). Among the samples with three different ratios, the equal ratio crystalline sized sample had the smallest particles, as evidenced by XRD, and was chosen as the optimized binary suspension matrix. Subsequently, based on the XRD data, among the three different ratio WHAC samples, WHAC (2 : 1) was selected due to its smallest crystalline size (see Table S1†). Then, 4%, 8%, and 12% w/v ratios of WHAC (2 : 1) were added separately to the MnO_2_/Fe_3_O_4_ (1 : 1) suspension with continuous stirring, and heated at 80 °C for approximately 60 min in the water bath to initiate the reduction process. Thereafter, each of the suspensions was blended for 50 min through stirring. After 50 min, a 2 M solution of NaOH was added dropwise to attain pH 11, and the solutions were left stirring for a further 15 min to regulate the solutions' acidity, signifying the formation of a precipitate. The separation of this precipitate was accomplished using a centrifuge operating at 3000 rpm for 5 min. After obtaining the precipitate, it underwent several rinses with ethanol–distilled water–ethanol in sequence to eliminate any impurities. The obtained ternary composites were dried overnight in an oven at 40 °C. To ensure the crystallinity and remove the low volatile impurities, all the dried powders were thoroughly heated in an oven at 200 °C for 6 h (see Scheme 1). Finally, the formed ultimate composites were labelled as MnO_2_/Fe_3_O_4_@4% AC, MnO_2_/Fe_3_O_4_@8% AC, and MnO_2_/Fe_3_O_4_@12% AC, and all were subjected to further characterization analysis and performance tests.

### 2.7. Characterization techniques

To determine the stable calcination temperature, thermogravimetric-differential thermal analysis (TGA-DTA) was carried out using a DTG-60H detector (Shimadzu, Japan) under nitrogen gas at a flow rate of 50 mL min^−1^ and run using an alumina cell. To determine the crystalline structure and average crystalline size of the prepared materials, X-ray diffraction (SHIMADZU, XRD-7000) was carried out at a voltage of 40 kV and a current of 30 mA using (CuKα = 1.5406 Å) radiation as an X-ray source, at a scanning rate of 2° min^−1^ in the 2*θ* range from 10° to 80°. The stretching and vibrational motions of the intermolecular bonding were characterized by Fourier transform infrared spectrometry (FTIR, PerkinElmer 65, BX spectrophotometer) in the wavenumber range of 4000–400 cm^−1^ with the samples prepared using KBr pellets. The specific surface area, pore size, and pore volume were calculated by the Brunauer–Emmett–Teller method using a BET analyzer (BET, Quantachrome NovaWin-Data Acquisition and Reduction for NOVA Instruments, version 11.0) *via* nitrogen adsorption/desorption isotherms at 77.35 K. The surface morphology and the chemical elements present were investigated by scanning electron microscopy (SEM) in conjunction with energy dispersive X-ray spectroscopy (SEM-EDX-EVO 18 model, low-vacuum facility, ALTO 1000 Cryo attachment). Furthermore, the microstructural surface topography, crystalline size distribution, and purity of the synthesized materials were elucidated by transmission electron microscopy (TEM), high-resolution transmission electron microscopy (HRTEM), and selected area electron diffraction (SAED) analyses using a JEOL TEM 2100 HRTEM instrument.

Electrochemical characterization measurements and performance tests were carried out in a standard three-electrode system using an IVIUM potentiostat, in which electrodes modified with the prepared materials served as the working electrode (WE), Pt wire as the counter electrode (CE), and Ag/AgCl as the reference electrode (RE) with 1 M KOH electrolyte solution. Cyclic voltammetry (CV) experiments were performed at varying scan rates from 10 to 100 mV s^−1^ in the 0–0.6 V range. The galvanostatic charge/discharge (GCD) tests were recorded at current densities of 1, 3, 5, and 7 A g^−1^ in the 0–0.6 V voltage window. For ethanol oxidation (direct fuel cell), 1 M KOH solution was used as an electrolyte in the −0.7 to 0.7 V voltage window. Linear sweep voltammetry (LSV) was carried out to measure the polarization curve in 1 M Na_2_SO_4_ and 1 M C_2_H_5_OH. Electrochemical impedance spectroscopy (EIS) was performed at 10 mV in the frequency range from 100 kHz to 0.01 Hz.

### 2.8. Preparation of the electrodes

Nickel (Ni) foam (2 cm × 1 cm) was used as a bare electrode current collector. To remove the surface oxides and other impurities, the rectangular-shaped Ni foam was etched using a 1 M HCl solution and left for 30 min.^2^ The etched Ni foam was then continuously rinsed with distilled water and ethanol and then dried at room temperature. Modification of the Ni foam using MnO_2_ (1 : 1), Fe_3_O_4_ (1 : 10), MnO_2_/Fe_3_O_4_ (1 : 1), MnO_2_/Fe_3_O_4_@4% AC, MnO_2_/Fe_3_O_4_@8% AC, and MnO_2_/Fe_3_O_4_@12% was performed following a previously reported method with slight modification.^21^ Briefly, 10 mg of PVA as a binder was added into a 100 mL beaker, and 10 mL of distilled water was added to this. To dissolve PVA completely, the solution was placed on a hot plate and continuously stirred at 45 °C for 2 h. After that, 90 mg of the active sample, such as MnO_2_ (1 : 1), Fe_3_O_4_ (1 : 10), MnO_2_/Fe_3_O_4_ (1 : 1), MnO_2_/Fe_3_O_4_@4% AC, MnO_2_/Fe_3_O_4_@8% AC, and MnO_2_/Fe_3_O_4_@12%, was thoroughly ground separately in a mortar to obtain a fine powder. Then, 250 μL of the already prepared PVA solution was added to each fine powder of the prepared materials and the mixture was subjected to 1 h sonication (for homogenization) in order to disperse the active NPs and composites. Finally, 100 μL of each sample slurry was pasted and on a 1 cm^2^ area of etched Ni-foam to homogeneously cover it utilizing a micropipette and this was then dried at 70 °C overnight. The active mass was determined from determining the difference in mass before and after the loading of the active materials using a four-digit electronic balance. The measured active mass for all modified electrode was close to 2 mg.

## 3 Result and discussion

### 3.1. Thermogravimetric-differential thermal analysis

**Fig. 1:**
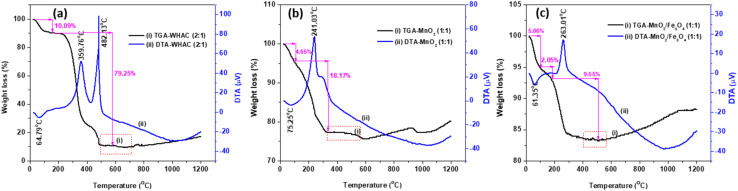
TGA-DTA thermograms of (a) WHAC (2 : 1), (b) MnO_2_ (1 : 1), and (c) MnO_2_/Fe_3_O_4_ (1 : 1).

The thermal analysis of all the activated carbon samples showed similar decomposition profiles. Fig. 1a shows the TGA-DTA thermograms for the prepared WHAC (2 : 1), while the thermograms for WHAC (1 : 1) and WHAC (1 : 2) are shown in Fig. S1.† The first mass loss stage for WHAC (2 : 1), which started from room temperature to a maximum exothermic peak at about 64.79 °C, involved the loss of the physically adsorbed water on WHAC (2 : 1), with an approximate weight loss of 10.09%. The greatest weight loss, at about 79.25%, involved two DTA endothermic peaks, one observed at about 359.76 °C, which was due to the evolution of carbon dioxide from the hemicelluloses, and another at 482.13 °C in the second stage, which was related to the combined decomposition of the carbon skeleton made of cellulose molecules^22^ and other surface groups.^23^ The third and last stage of the decomposition occurred in the range between 400 °C and 500 °C, and was due to the degradation of lignin.^24,25^ Above 500 °C, the decomposition of oxygenated surface groups, such as carbonyl, quinones, hydroquinone, and other structures would occur.^23^ At 500 °C not much weight loss occurred, and so 500 °C was selected as the highest stable carbonization temperature for AC preparation. Fig. 1b indicates the thermal properties of the as-synthesised MnO_2_ (1 : 1). A 4.65% weight loss with an exothermic peak at about 75.25 °C was observed, which indicated the loss of physically adsorbed water molecules and organic compounds during the synthesis. A strong peak with a sharp endothermic peak was found at about 241.03 °C in the DTA curve with a 18.17% mass loss in the TGA, which was due to the removal of alkyl groups or carbon residues, as well as loss of the interlayer water/hydroxyl groups/decomposition of high valence MnO_*x*_, and formation of MnO_2_.^26^ Thus, 400 °C was chosen as the highest stable temperature for the delta-phase MnO_2_. However, a small weight loss in the 400–500 °C temperature range was observed in the TGA, which could be associated with the loss of oxygen from the MnO_2_ lattice. The unstable curve above 500 °C was attributed to the decomposition of MnO_2_ into other phases.^27^ The thermal stability of MnO_2_/Fe_3_O_4_ (1 : 1) is shown in Fig. 1c. As indicated, there were three stages of decomposition correlated with weight loss. The first stage with DTA was found at 61.35 °C with a mass loss of 6.06%, which was related to the loss of adsorbed water and other volatile organic compounds during the synthesis. The second stage in the range of 100–200 °C was related to the removal of crystalline water, with a 2.05% mass loss. The third stage of weight loss, of about 9.55% within the temperature range of 200–400 °C, could be ascribed to the crystallization turning to medium. The highest stable temperature was 450 °C as there was no weight loss at this point, while above 450 °C, there was phase transformation with loss of oxygen from MnO_2_ to Mn_2_O_3_,^28^ corresponding to the thermal decomposition of the crystal phase with the transformation of Fe_3_O_4_ to γ-Fe_2_O_3_.^29^

### 3.2. XRD analysis

**Fig. 2:**
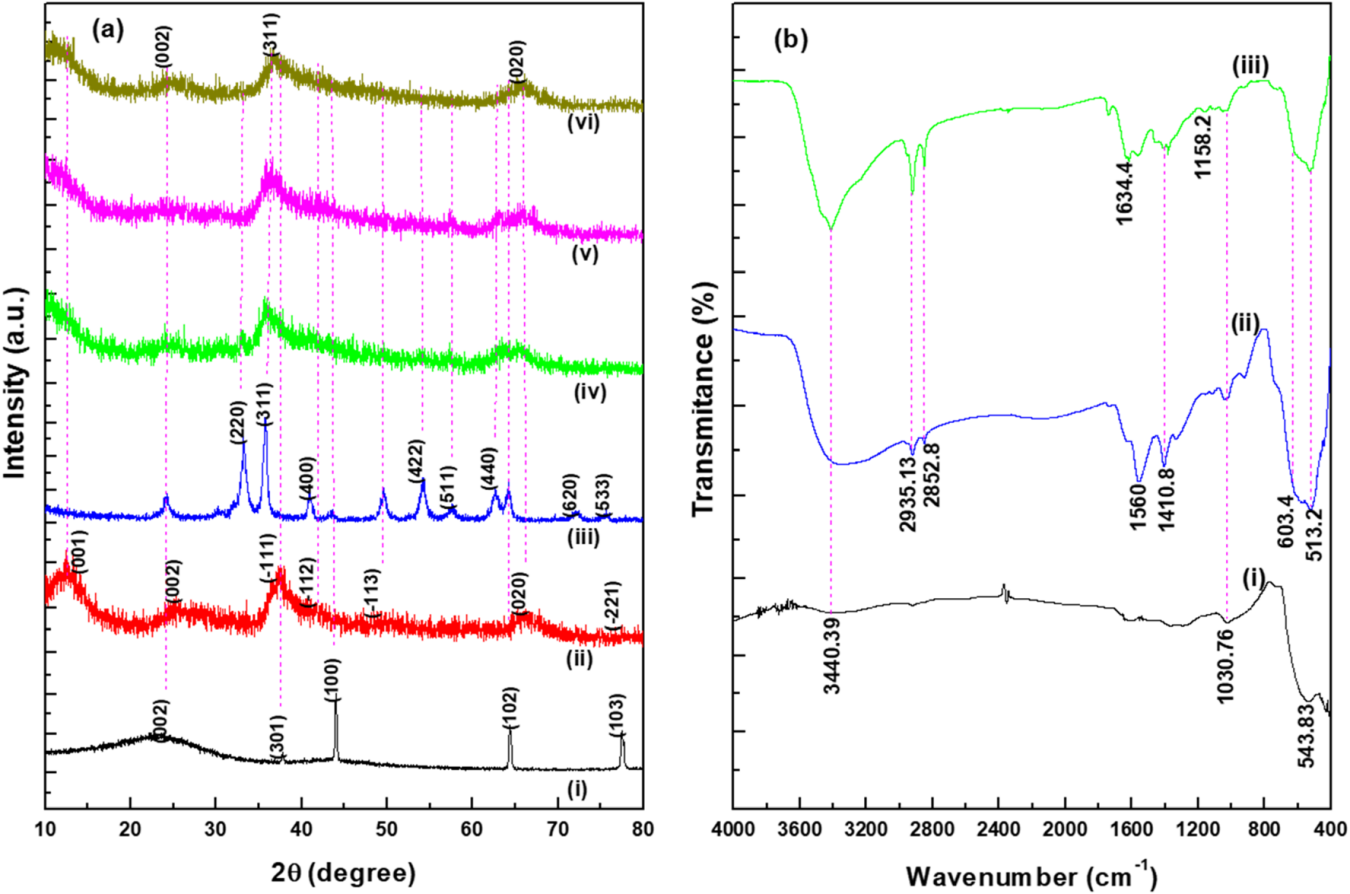
XRD patterns (a) of (i) WHAC (2 : 1), (ii) MnO_2_ (1 : 1), (iii) MnO_2_/Fe_3_O_4_ (1 : 1), (iv) MnO_2_/Fe_3_O_4_ (1 : 1)@4% AC, (v) MnO_2_/Fe_3_O_4_ (1 : 1)@8% AC, (vi) MnO_2_/Fe_3_O_4_ (1 : 1)@12% AC. FTIR spectra (b) for (i) MnO_2_ (1 : 1), (ii) MnO_2_/Fe_3_O_4_ (1 : 1), and (iii) MnO_2_/Fe_3_O_4_ (1 : 1)@8% AC.

The XRD patterns of the synthesized WHAC (2 : 1), MnO_2_ (1 : 1), MnO_2_/Fe_3_O_4_ (1 : 1), MnO_2_/Fe_3_O_4_ (1 : 1)@4% AC, MnO_2_/Fe_3_O_4_ (1 : 1)@8% AC, and MnO_2_/Fe_3_O_4_ (1 : 1)@12% AC samples were recorded in the 2*θ* range between 10°–80°, as shown in Fig. 2a. It was observed that for the WHAC (2 : 1) sample (Fig. 2a(i)), the main intensity peaks could be observed at 2*θ* = 23.7°, 37.7°, 44.1°, 64.4°, and 77.6°, corresponding to the (002), (301), (100), (102), and (103) crystal planes, respectively; for comparison, the XRD patterns of WHAC (1 : 2), WHAC (1 : 1), and WHAC (2 : 1) are shown in Fig. S2.† However, due to its smallest crystalline size, WHAC (2 : 1) was chosen as the optimized AC material. Hence, the broad diffraction peak at 2*θ* = 23.7°, corresponding with the (002) plane, indicated the presence of a disordered graphite-like carbon structure, while the rest of the sharp peaks corresponding to the (301), (100), (102), and (103) crystal planes provided evidence of a graphite carbon structure with a high degree of graphitization formation.^30^ As shown in Fig. 2a(ii), the main diffraction peaks found at 2*θ* = 12.34°, 24.42°, 37.90°, and 66.12°, corresponding with the (001), (002), (−111), and (020) crystal planes, respectively, showed similar patterns to the K-birnessite-type manganese dioxide crystal (*δ*-MnO_2_), in accordance with JCPDS card no. 80-1098.^31,32^ Besides, the diffractogram presented a significantly high signal-to-noise ratio. This was also a consequence of the powder diffraction technique, where many reflections with low resolution are available.^33^Fig. 2a(iii) shows the XRD patterns of MnO_2_/Fe_3_O_4_ (1 : 1). The characteristic peaks found at 2*θ* = 33.14°, 35.76°, 41.18°, 54.14°, 57.54°, 62.78°, 71.98°, and 75.56°, corresponding with the (220), (311), (400), (422), (511), (440), (620), (533) crystal planes, respectively, concurred with the crystal structural pattern of magnetite (JCPDS no. 96-900-7645).^34^ The occurrence of an additional pattern with minor peak shifts in MnO_2_/Fe_3_O_4_ (1 : 1) with diffraction angles of 24.26°, 49.78°, and 64.18° might indicate the presence and proper development of MnO_2_. Fig. 2a(iv)–(vi) present the XRD patterns of MnO_2_/Fe_3_O_4_ (1 : 1)@4% AC, MnO_2_/Fe_3_O_4_ (1 : 1)@8% AC, and MnO_2_/Fe_3_O_4_ (1 : 1)@12% AC. It is evident from the XRD patterns that the intensity of the peaks related to the MnO_2_/Fe_3_O_4_ (1 : 1) phase changed as the AC content increased in the composite samples. However, no other peak related to any other phase was seen, which confirmed the phase purity of the active materials. Their average crystalline sizes (*D*) are shown in Table S1.† Among the ternary composites, the average size of MnO_2_/Fe_3_O_4_ (1 : 1)@8% AC was found to be small, with a value of 7.65 nm, and this was selected as the optimized ternary composite for further study. This value was determined from the full width at half maxima (*β*) and the Bragg angle (*θ*) of the (002), (311), and (020) reflections using Scherrer's equation, as given below:^34^1
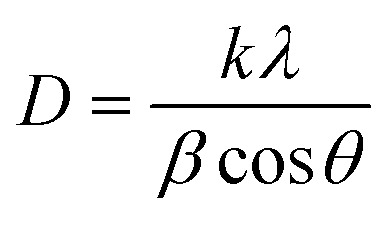
where *D* refers to the mean diameter of the crystalline domain of the particles, *k* is the constant proportionality parameter (assumed *k* = 0.89), and *λ* is the wavelength of X-ray radiation (*λ* = 0.15406 nm).

### 3.3. FTIR analysis

**Fig. 3:**
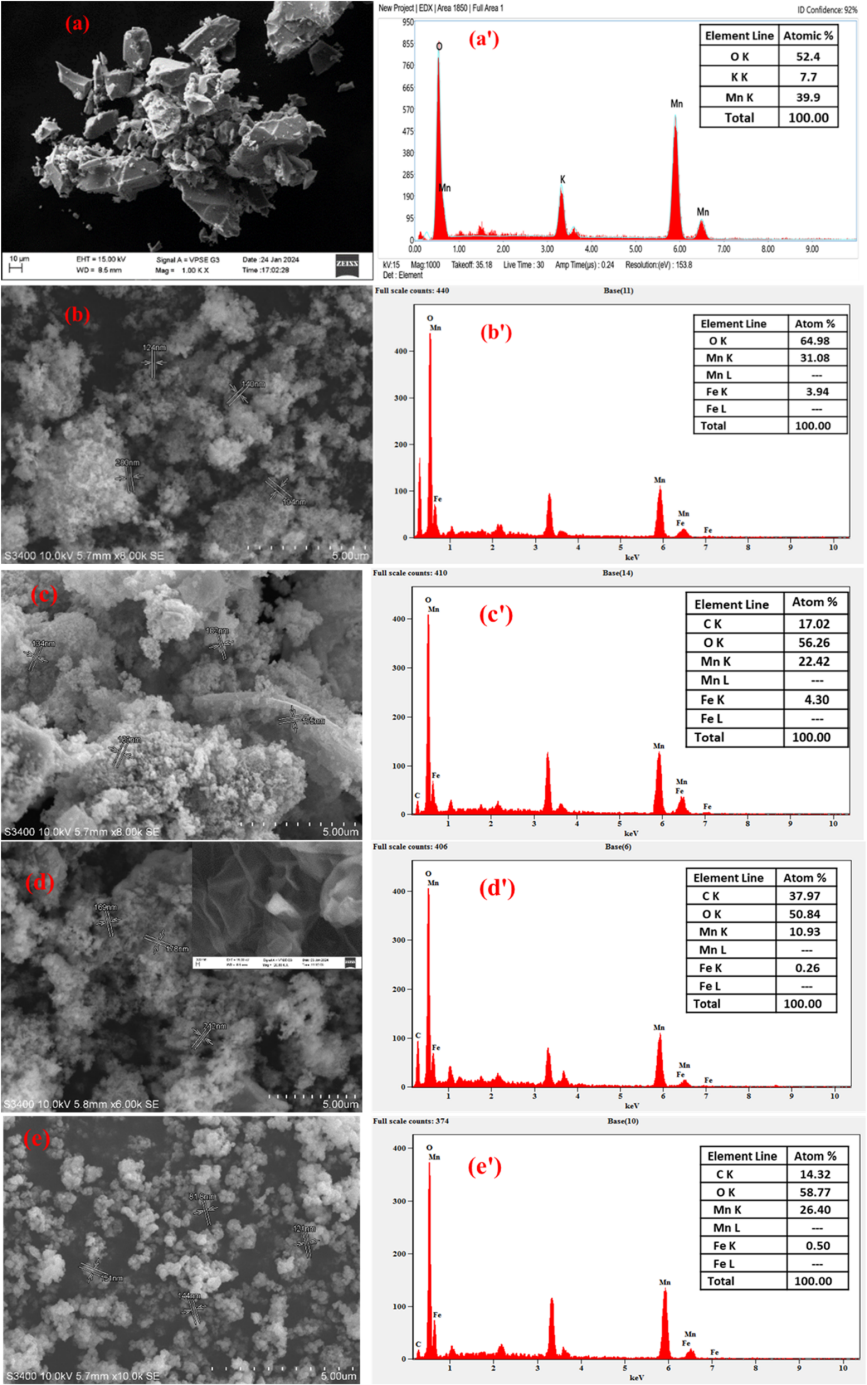
SEM images (a–e) and EDX spectra (a’–e’) of MnO_2_ (1 : 1), MnO_2_/Fe_3_O_4_ (1 : 1), MnO_2_/Fe_3_O_4_ (1 : 1)@4% AC, MnO_2_/Fe_3_O_4_ (1 : 1)@8% AC, and MnO_2_/Fe_3_O_4_ (1 : 1)@12% AC.

The shift in the spectra and reduction and disappearance of certain peaks were used to assess whether there was an effect from the NPs integration on the composites as well as to identify the surface functional groups on the prepared samples. Fig. 2b presents the FTIR spectra of the MnO_2_ (1 : 1), MnO_2_/Fe_3_O_4_ (1 : 1), and MnO_2_/Fe_3_O_4_ (1 : 1)@8% AC samples. The broad peak observed at around 3440.39 cm^−1^ in all the spectra was associated with the bands of the O–H group due to the vibration of surface-adsorbed water molecules. Another obvious peak found at about 2935.13 cm^−1^, as shown in Fig. 2b(ii) and (iii), was due to the presence of aliphatic C–H stretching of CH, CH_2_, and CH_3_ groups, while those peaks related to the organic compounds were not found in the pure MnO_2_ spectrum at 2935.13 and 2852 cm^−1^ due to their removal by calcination, as shown in Fig. 3b(i). The observed peak at about 1634.4 cm^−1^ corresponded to the C=O stretching of carboxylic acids.^35^ The weak peaks found at 1410.8 cm^−1^ were assigned to C–H asymmetric and symmetric bending while the peak at 1158 cm^−1^ indicated C=C bond vibration in carbon.^36^ For additional detail, the pure FTIR spectrum of WHAC (2 : 1) is shown in Fig. S3† and was reported in our previous published work.^15^ As shown in Fig. 2b(i), the peak at about 543.83 cm^−1^ was due to the stretching of Mn–O bonding in the *δ*-MnO_2_ crystal, corresponding to the modes from the octahedral layers in the birnessite structure,^31^ which was also consistent with the XRD patterns. The Fe–O–C band illustrated near 1030.76 cm^−1^ showed that the sample properly integrated the AC with Fe_3_O_4_.^37^ The peak close to 603.4 cm^−1^ was due to the asymmetric bending vibration of the Fe–O bond in octahedral Fe_3_O_4_.^34^ So, the formation of weak in absorption and a new shift in wavenumber at about 513.2 cm^−1^ may be some new bond generated between MnO_2_/Fe_3_O_4_ (1 : 1) and AC, which was also supported by XRD results.

### 3.4. Surface area and pore volume analysis

The multipoint specific surface area, HK pore size, and NLDFT pore volume computations are shown in Table 1. The specific surface areas of MnO_2_ (1 : 1), Fe_3_O_4_ (1 : 10), MnO_2_/Fe_3_O_4_ (1 : 1), MnO_2_/Fe_3_O_4_ (1 : 1)@4% AC, MnO_2_/Fe_3_O_4_ (1 : 1)@8% AC, and MnO_2_/Fe_3_O_4_ (1 : 1)@12% AC were calculated using a BET analyzer (Quantachrome NovaWin-Data Acquisition and Reduction for NOVA Instruments, version 11.0) *via* nitrogen adsorption/desorption isotherms at 77.35 K. The total porous information of the prepared materials was under the range of microspores (*i.e.*, <2 nm), as calculated from the BET analysis, and detailed data are summarized in Table 1. The total volumes of MnO_2_ (1 : 1), Fe_3_O_4_ (1 : 10), MnO_2_/Fe_3_O_4_ (1 : 1), MnO_2_/Fe_3_O_4_ (1 : 1)@4% AC, MnO_2_/Fe_3_O_4_ (1 : 1)@8% AC, and MnO_2_/Fe_3_O_4_ (1 : 1)@12% AC were found to be 0.1791, 0.1837, 0.1386, 0.1570, 0.1306, and 0.1345, respectively. The specific surface areas estimated cumulative pore volume, and average pore radius of pure AC were found to be 522.499 m^2^ g^−1^, 0.16 cm^3^ g^−1^ and 0.92 nm, respectively,^15^ while the specific surface areas of MnO_2_ (1 : 1), Fe_3_O_4_ (1 : 10), MnO_2_/Fe_3_O_4_ (1 : 1), MnO_2_/Fe_3_O_4_ (1 : 1)@4% AC, MnO_2_/Fe_3_O_4_ (1 : 1)@8% AC, and MnO_2_/Fe_3_O_4_ (1 : 1)@12% AC were determined to be 498.281, 557.151, 547.922, 539.288, 545.163, and 524.683 m^2^ g^−1^, respectively. In summary, the specific surface area of MnO_2_ (1 : 1) was significantly higher than that of commercial MnO_2_ produced by traditional methods.^38,39^ This increases the specific surface area of the binary composite MnO_2_/Fe_3_O_4_ (1 : 1). However, the specific surface areas of the ternary composites MnO_2_/Fe_3_O_4_ (1 : 1)@4% AC, MnO_2_/Fe_3_O_4_ (1 : 1)@8% AC, and MnO_2_/Fe_3_O_4_ (1 : 1)@12% AC were found to be lower compared to the bare Fe_3_O_4_ (1 : 10) and MnO_2_/Fe_3_O_4_ (1 : 1). This might be due to the low content of AC and blocking of the pore structure by MnO_2_ (1 : 1), as evidenced in similar earlier reports.^40^ Again, from the ternary composites with fixing the MnO_2_/Fe_3_O_4_ (1 : 1) mass, when the amount of AC increased from 4 wt% to 8 wt%, the specific surface area increased from 539.288 to 545.163 m^2^ g^−1^ due to the higher surface area of MnO_2_/Fe_3_O_4_ (1 : 1) and the formation of a high concentration of AC in MnO_2_/Fe_3_O_4_ (1 : 1)@4% AC and MnO_2_/Fe_3_O_4_ (1 : 1)@8% AC. It could be seen that the pore volume decreased as the mass of AC increased in the formed composites (MnO_2_/Fe_3_O_4_ (1 : 1)@12% AC), probably due to blocking of the cluster-type low specific surface area of *δ*-MnO_2_. It is known that increasing the mass of AC in a metal oxide/activated carbon composite decreases the surface area per unit mass, while adding more AC provides more potential adsorption sites.^41^ Later, when the mass of AC was 8 wt%, the surface area of MnO_2_/Fe_3_O_4_ (1 : 1)@12% AC was reduced to 524.683 m^2^ g^−1^, which might be due to providing additional bulk material that could physically block access to existing pores, effectively “covering up” some of the available surface area within the composite material; thus, essentially, the added mass would not contribute proportionally to the overall accessible surface area.^42^

**Table 1:** Summary of the porosity parameters of the synthesized NPs and composites

Sample code	Specific surface area (m^2^ g^−1^)	Pore size (nm)	Pore volume (cm^3^ g^−1^)
MnO_2_ (1 : 1)	498.281	0.8837	0.1791
Fe_3_O_4_ (1 : 10)	557.151	0.1838	0.1837
MnO_2_/Fe_3_O_4_ (1 : 1)	547.922	0.8787	0.1386
MnO_2_/Fe_3_O_4_ (1 : 1)@4%AC	539.288	0.1838	0.1570
MnO_2_/Fe_3_O_4_ (1 : 1)@8%AC	545.163	0.8437	0.1306
MnO_2_/Fe_3_O_4_ (1 : 1)@12%AC	524.683	0.8812	0.1345

### 3.5. Surface morphology measurements

#### 3.5.1. SEM-EDX analysis

The surface morphology of *δ*-MnO_2_ synthesized through a green method using WH leaf extract as a reducing agent was elucidated by SEM. Fig. 3a shows it had a rough and irregular surface with the presence of cavities. Further, the existence of small heterogeneous particulates on the surface might be related to the growth of small-sized MnO_2_ particles, as evidenced in the literature.^43^ Its elemental composition was dominated by Mn and O with a total atomic percentage of 92.3%, as shown in Fig. 3a′. The SEM-EDX images, shown in Fig. 3b, b′, e and e′, indicate the morphology and elemental composition of the prepared composites. It could be observed from the images for the binary MnO_2_/Fe_3_O_4_ (1 : 1) composites, shown in Fig. 3b, to those of the ternary composites, such as MnO_2_/Fe_3_O_4_ (1 : 1)@4% AC, MnO_2_/Fe_3_O_4_ (1 : 1)@8% AC, and MnO_2_/Fe_3_O_4_ (1 : 1)@12% AC, that there were irregular particles of various sizes with minor distinct surface morphology differences (Fig. 3c–e). In particular, the binary MnO_2_/Fe_3_O_4_ (1 : 1) composites created an interconnected network resembling a highly porous thin sheet of AC (see the inset Fig. 3d). As a result, the pore-size distribution, pore volume, and specific surface area of the ternary composites, such as MnO_2_/Fe_3_O_4_ (1 : 1)@4% AC, MnO_2_/Fe_3_O_4_ (1 : 1)@8% AC, and MnO_2_/Fe_3_O_4_ (1 : 1)@12% AC, were clearly modified, as evidenced from the BET and XRD data. EDX analysis of the binary MnO_2_/Fe_3_O_4_ (1 : 1) composites consisting of only Mn, Fe, and O with 100% atomic composition showed the pure formation of binary composites. However, the atomic percentage of Mn was reduced in the binary composites due to the increase in atomic % of Fe (see Fig. 3b′). The presence of two metals (Mn and Fe) and two non-metals (C and O), as shown in Fig. 3c′–e′, indicated the formation of ternary composites in different ranges. The relative variation in the wider range of elemental compositions for each element revealed that the heterogeneity of the composites might be due to the dispersion techniques employed during the preparation method, as evidenced in other reports.^44^ From the SEM micrographs and other previous literature reports, the observed porosity could be quite beneficial to providing the maximum active sites for electrochemical reactions.^45^

#### 3.5.2. TEM-HRTEM-SAED analysis

**Fig. 4:**
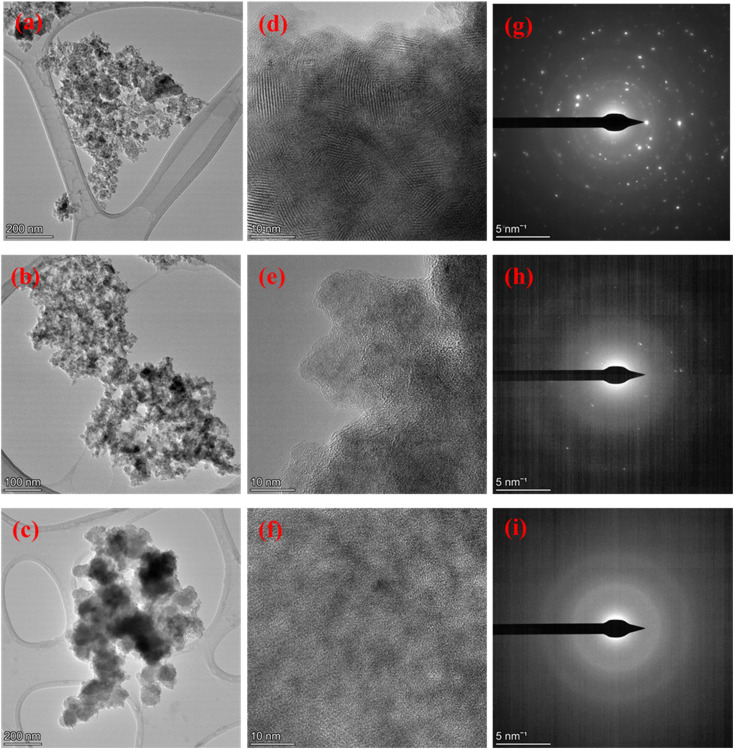
TEM images (a–c), HRTEM micrographs (d–f), and SAED patterns (g–i) of MnO_2_ (1 : 1), MnO_2_/Fe_3_O_4_ (1 : 1), and, MnO_2_/Fe_3_O_4_ (1 : 1)@8% AC.

The TEM image of pure *δ*-MnO_2_ is shown in Fig. 4a, which displays its quite irregular porous surface with the presence of cavities almost throughout, including internally. The surface observed at a magnification of 200 nm appeared to be very rough, as clearly observed from the SEM image. The presence of lattice fringes in the HRTEM micrograph, as shown in Fig. 4d, suggested that *δ*-MnO_2_ had good crystallinity. Such crystallinity was further supported by the clear, bright circular spots in its SAED pattern, as indicated in Fig. 4g. In the case of the MnO_2_/Fe_3_O_4_ (1 : 1) binary composites shown in Fig. 4b, broad halos could be observed together with diffraction signals due to the dominancy of the magnetite characteristic of the binary composites and resulting from the amorphous coating of the composites by Fe_3_O_4_.^46^ The incorporation of *δ*-MnO_2_ by Fe_3_O_4_, as shown in the HRTEM image in Fig. 4e, indicates that the composites had low crystallinity with different crystallographic orientations due to the incorporation of amorphous magnetite nanostructures.^8^ The low crystallinity of the binary composites, as shown in Fig. 4h, was also supported by the few circular spots in the SAED pattern. Finally, from the TEM images shown in Fig. 4c, a loosely arranged spherical-like structure with the absence of lattice fringes could be seen in the HRTEM image in Fig. 4f, with no white circular spots, as evidenced from the SAED pattern shown in Fig. 4i. From such evidence, it could be concluded that binary composites of MnO_2_/Fe_3_O_4_ (1 : 1) had apparently grown on the surface of the porous AC and formed the ternary MnO_2_/Fe_3_O_4_ (1 : 1)@8% AC.

### 3.6. Electrochemical measurements

#### 3.6.1. Charge-storage analysis

**Fig. 5:**
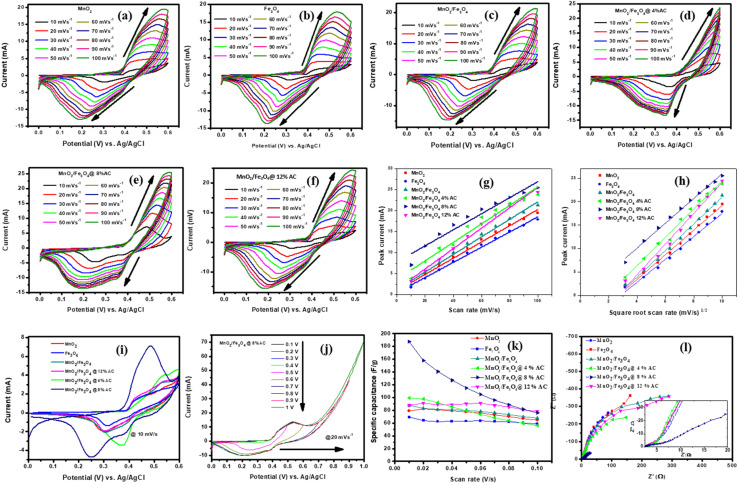
CV curves (a–f) under different scan rates (10–100 mVs^−1^), peak current *versus* scan rates (g); peak current *versus* square root of the scan rates (h); combined CV curves at 10 mV s^−1^ (i); CV curves under different potential windows for MnO_2_/Fe_3_O_4_ (1 : 1)@8% AC at 20 mV s^−1^ (j); specific capacitance *versus* scan rates (k); Nyquist plots for pure MnO_2_ (1 : 1) and Fe_3_O_4_ (1 : 1); and MnO_2_/Fe_3_O_4_ (1 : 1), MnO_2_/Fe_3_O_4_ (1 : 1)@4% AC, MnO_2_/Fe_3_O_4_ (1 : 1)@8% AC, and MnO_2_/Fe_3_O_4_ (1 : 1)@12% AC composites (l).

The electrochemical performances of the pure and composite-modified electrodes were investigated using CV, GCD, and EIS techniques. The charge-storage mechanism and charge-storage ability were elucidated by CV. Voltammograms of the pure and composite materials are shown in Fig. 5a–f collected in the scan rate range from 10–100 mVs^−1^. The redox peak for each cycle indicated the pseudocapacitive charge-storage behavior of the material due to faradaic redox reactions. When the scan rate was increased, the symmetry of each cycle's peaks did not change, indicating the effectiveness of the charge-storage mechanism and the stability of the materials.^47^ The shift in positive potential for the anodic peak and shift in negative potential for the cathodic peak with increasing the scan rate indicated the role of the polarization effect and the ideal capacitive behavior of the electrodes. Hence, the voltammogram profile for each sample reflected clear redox peaks due to the faradaic reactions of the supercapacitors, with a dominant pseudocapacitive nature.^48–50^ The surface area under the voltammogram curve for the MnO_2_/Fe_3_O_4_ (1 : 1)@4% AC, MnO_2_/Fe_3_O_4_ (1 : 1)@8% AC, and MnO_2_/Fe_3_O_4_ (1 : 1)@12% AC electrodes was greater than that of MnO_2_ (1 : 1) and Fe_3_O_4_ (1 : 1), and MnO_2_/Fe_3_O_4_ (1 : 1) due to the enhanced ion- and automatic charge-transport rates resulting from the good synergistic effect between MnO_2_/Fe_3_O_4_ (1 : 1) and AC, which could be seen at higher potentials through amplification of the highest current values with faster scan rates. Even the shapes of the CV curves of MnO_2_/Fe_3_O_4_ (1 : 1)@4% AC, MnO_2_/Fe_3_O_4_ (1 : 1)@8% AC, and MnO_2_/Fe_3_O_4_ (1 : 1)@12% AC were more significant than those of pure MnO_2_ (1 : 1), Fe_3_O_4_ (1 : 1), and MnO_2_/Fe_3_O_4_ (1 : 1), indicating the higher charge-storage behaviors of MnO_2_/Fe_3_O_4_ (1 : 1)@4% AC, MnO_2_/Fe_3_O_4_ (1 : 1)@8% AC, and MnO_2_/Fe_3_O_4_ (1 : 1)@12% AC than the others. Under different scan rates, a slight shift in the redox current peaks to upper and lower potentials was detectable, respectively, confirming the electrode's resistance and that the reaction processes were kinetics-controlled.^45^ From the peak current *versus* scan rates plots shown in Fig. 5g and the correlation coefficients found in Table 2, a linear relationship could be observed. Hence, from Table 2, as the scan rates increased, the peak current also increased proportionally. This is because at higher scan rates, there was less time for ions to fully participate in charge-transfer reactions at the electrode surface, leading to larger current spikes at the peak potential.^51^

**Table 2:** Summary of the correlation coefficient, anodic peak current *versus* scan rates, and equations for the anodic peak current *versus* square root of the scan rates^a^

Catalysts	*I*_pa_ *vs. v* ^1/2^		*I*_pa_ *vs. v*	
	Equation	*R* ^2^	Equation	*R* ^2^
MnO_2_ (1 : 1)	*y* = 2.61758*x* − 6.99907	0.99526	*y* = 0.19331*x* + 0.967	0.99076
Fe_3_O_4_ (1 : 1)	*y* = 2.43902*x* − 6.91574	0.99204	*y* = 0.18061*x* + 0.48033	0.99353
MnO_2_/Fe_3_O_4_ (1 : 1)	*y* = 2.82865*x* − 7.35992	0.99516	*y* = 0.20897*x* + 1.2448	0.99138
MnO_2_/Fe_3_O_4_ (1 : 1)@4%AC	*y* = 3.01493*x* − 5.65825	0.98823	*y* = 0.22008*x* + 3.65847	0.95830
MnO_2_/Fe_3_O_4_ (1 : 1)@8%AC	*y* = 2.63671*x* − 0.40737	0.99406	*y* = 0.191*x* + 7.8216	0.94737
MnO_2_/Fe_3_O_4_ (1 : 1)@12%AC	*y* = 3.27682*x* − 8.64951	0.98869	*y* = 0.24266*x* + 1.28653	0.99027

a *I*_pa_ = anodic peak current, *y* = *I*_pa_, and *v*^1/2^ = *x*

For all the voltammogram curves, it could be seen that the redox current gradually increased with increasing the scan rate, indicating the ideal capacitive behavior of all the electrodes. In addition, the shifting of the anodic peaks and cathodic peaks toward the positive and the negative potentials, respectively, verified the reactions were quasi-reversible. The anodic peak current *versus* square root of the scan rate plots shown in Fig. 5h and the linear relationship dominance between the anodic peak currents and the square root of the scan rates found in Table 2 for the pure and composite-modified electrodes also supported the occurrence of quasi–reversible reactions followed by a diffusion-controlled process on the electrode surfaces.

The CV curves for the pure and different composites at a common scan rate of 10 mV s^−1^ are shown in Fig. 5i. They display a pair of oxidation and reduction peaks that correspond to the significant capacitance caused by their pseudocapacitive behaviour. An excellent reversibility reaction with a higher oxidation and reduction current for MnO_2_/Fe_3_O_4_ (1 : 1) in the presence of 12% AC is observed due to modification in specific surface area and pore volume under a curve of MnO_2_/Fe_3_O_4_ (1 : 1)@8%AC electrode than that of the other. When MnO_2_/Fe_3_O_4_ (1 : 1) aligned with biomass-based AC energy storage devices, due to their porous nature has an advantage of accommodating of electrolyte ions that diffuse inside their channels when they composite with pseudocapacitor materials.^52^ The higher potential windows lead to a decrease in the total charge (*Q*_t_) of the electrode, and the CV profile indicates they maintained resistive behaviors close to non-ideality (non-rectangular shape), as shown in Fig. 5j. A potential window from 0.0 to 1.0 V was chosen to identify the electrode oxidation and reduction processes. So, the window between 0.0 V and close to 0.6 V was chosen to ensure the maximum faradaic charges. The lower potential and higher than 0.6 V may lead to irreversible reduction of Mn^4+/2+^, Fe^3+/2+^ and to oxygen evolution.^53,54^ The values of the specific capacitance for each pure and composite-modified electrode were calculated using eqn (2).^45^2
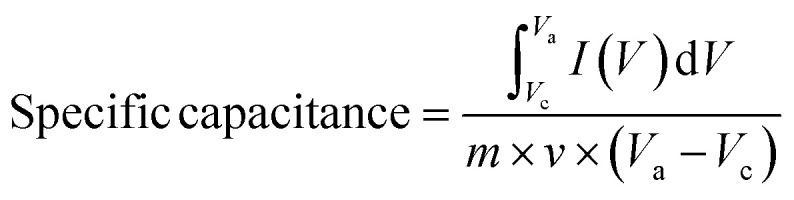
where *v* is the scan rate (V s^−1^), *m* is the measured mass (g), *I* is the current (A), and (*V*_a_–*V*_c_) is the potential window (V). Fig. 5k summarizes the relationship between the specific capacitance and the scan rate for the pure and composite-modified electrodes. The specific capacitance decreased as the scan rate increased in most electrodes due to the limitations of electrolyte ion diffusion within the porous structure of the materials, which also restricts the charge-storage capacity at faster charge–discharge cycles. At low scan rates, the electrolyte ions have sufficient time to access the entire surface area, while at higher scan rates, electrolyte ion-diffusion is limited, causing a drop in the charge-storage capacity. Table 3 presents the specific capacitance values for the different pure and composite-modified electrodes at different scan rates. It could be observed that the modification of MnO_2_/Fe_3_O_4_ (1 : 1) with AC led to an enhanced specific capacitance. Among the modified materials, MnO_2_/Fe_3_O_4_ (1 : 1) with 8% AC exhibited the highest specific capacitance, which resulted in its best charge-storage capability performance. The increase in AC content from 4% AC to 8% AC at lower scan rates led to an enhanced charge-storage capacity due to the improvement in the specific surface area and pore volume, along with improved and low charge-transfer resistance and the best conductivity of the electrodes, as shown in the EIS curves (see Fig. 5l). However, when the mass loading of AC reached 12% AC, the specific capacitance declined, even at higher scan rates, due to a variety of controlling factors, such as pore size distribution, the presence of a combination of micro-and mesoporous nature, as well as the specific surface area nature.^55,56^ The enhanced specific capacitance demonstrated by the MnO_2_/Fe_3_O_4_ (1 : 1)@8%AC electrode clearly led to its best performance for energy storage compared to the others. From these findings, an optimized mass loading of biomass AC incorporated with faradaic types of MnO_2_/Fe_3_O_4_ (1 : 1) binary composites emphasizes the significance accumulating an outstanding energy storage devices.

**Table 3:** Summary of the specific capacitances of pure MnO_2_ (1 : 1), Fe_3_O_4_ (1 : 1), and MnO_2_/Fe_3_O_4_ (1 : 1), MnO_2_/Fe_3_O_4_ (1 : 1)@4% AC, MnO_2_/Fe_3_O_4_ (1 : 1)@8% AC, and MnO_2_/Fe_3_O_4_ (1 : 1)@12% AC electrodes at different scan rates

Scan rates	MnO_2_	Fe_3_O_4_	MnO_2_/Fe_3_O_4_	MnO_2_/Fe_3_O_4_@4%AC	MnO_2_/Fe_3_O_4_ at 8%AC	MnO_2_/Fe_3_O_4_ at 12%AC
10	79.334	69.125	87.518	99.518	187.409	87.612
20	82.298	63.944	83.116	97.807	157.908	90.751
30	80.705	62.504	81.088	92.063	140.497	89.057
40	79.213	62.792	80.922	85.667	126.876	88.358
50	77.186	62.985	79.600	79.247	114.809	90.132
60	75.490	63.511	78.117	73.779	104.869	91.131
70	72.827	62.475	75.573	68.306	95.9109	88.009
80	70.441	61.842	73.235	64.010	88.574	85.118
90	66.993	60.085	69.879	59.403	81.551	80.951
100	64.540	58.993	67.656	55.891	76.130	77.980

**Fig. 6:**
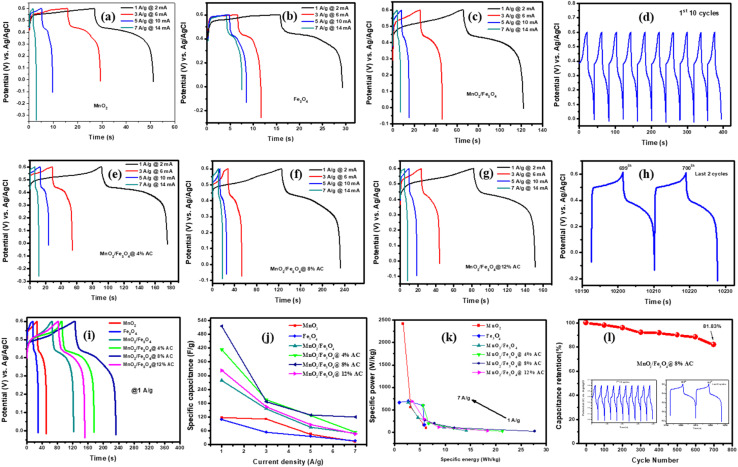
GCD curves (a–c and e–g) at different anodic and cathodic currents (2–10 mA), GCD curves of MnO_2_/Fe_3_O_4_ (1 : 1)@8% AC for the first 10 cycles (d) and last two cycles as the 699th and 700th cycles (h), GCD curves of different pure and composite modified electrodes at 1 A g^−1^ (i), specific capacitances for different electrodes at different current densities (j), Ragone plots comparing the specific power with the specific energy for different electrodes at different current densities (k), and capacitance retention of MnO_2_/Fe_3_O_4_ (1 : 1)@8% AC after 700 cycles (l).

Fig. 6a–c and e–g present the GCD curves for the pure and composite-modified electrodes at current densities of 1, 3, 5, and 7 A g^−1^. In all the curves, the discharge time was reduced when the current density was increased in all the electrode devices. This might be due to the electrolyte ion-diffusion effect (*i.e.*, the slow mass-transport process). That is, at higher current densities, KOH ions do not have enough time for their diffusion into the electrode's inner pores, and this causes the charge-storage capacity (specific capacitance) of the provided electrodes (s) to be reduced.^57^Fig. 6d shows the non-triangular GCD curves of MnO_2_/Fe_3_O_4_ (1 : 1)@8% AC for the first 10 cycles at a current density of 1 A g^−1^, which were due to the non-ideal dominancy with nonelectrostatic behaviors. Again for the last two consecutive cycles formed at the 699th and 700th cycles, as shown in Fig. 6h, indicated the nonelectrostatic behavior of MnO_2_/Fe_3_O_4_ (1 : 1)@8% AC electrode was indicated. The GCD curves shown in Fig. 6i indicated there was an increase in discharge time from the pure to composite-modified electrodes at a current density of 1 A g^−1^. From AC-modified MnO_2_/Fe_3_O_4_ (1 : 1) electrodes, the discharge time was elucidated due to the best synergistic effect between the carbon and metallic oxide framework, but the curve is a non-triangle GCD and nonelectrostatic behavior, as evidenced by other studies.^58^ The nearly symmetrical, not fully symmetrical, triangle found on the electrode indicated pseudo-faradaic reactions occurred on the devices.^59^ Therefore, in proportion, the dependence of the specific capacitance with a lower current density at 1 A g^−1^ was best in the MnO_2_/Fe_3_O_4_ (1 : 1)@8% AC electrode, as shown in Fig. 6j. From Table 4, it could be observed that the MnO_2_/Fe_3_O_4_ (1 : 1)@8% AC electrode possessed a remarkable specific capacitance, with a value of 515.113 F g^−1^ at 1 A g^−1^, which confirmed this was the optimum electrode that could best enable storing charge compared to the other supercapacitors.

**Table 4:** Summary of the specific capacitance, specific energy, and specific power for MnO_2_ (1 : 1), Fe_3_O_4_ (1 : 1), and MnO_2_/Fe_3_O_4_ (1 : 1), MnO_2_/Fe_3_O_4_ (1 : 1)@4% AC, MnO_2_/Fe_3_O_4_ (1 : 1)@8% AC, and MnO_2_/Fe_3_O_4_ (1 : 1)@12% AC at different current densities

Sample	Current density (A g^−1^)	Discharge time (s)	Specific capacitance (F g^−1^)	Specific energy (W h kg^−1^)	Specific power (W kg^−1^)
MnO_2_ (1 : 1)	1	24.2	117.857	6.211	91.636
	3	13.6	111.475	5.761	161.471
	5	4.6	45.480	3.166	554.087
	7	1.4	13.390	1.647	2419.714
Fe_3_O_4_ (1 : 1)	1	13.6	109.677	5.856	164.118
	3	5.2	54.292	5.603	596.769
	5	3.8	36.438	2.697	691.579
	7	3.4	16.294	0.887	662.824
MnO_2_/Fe_3_O_4_ (1 : 1)	1	56.2	278.678	14.167	38.754
	3	21	156.483	9.785	115.029
	5	7.2	76.248	4.627	330.500
	7	3.4	48.882	2.661	662.824
MnO_2_/Fe_3_O_4_ (1 : 1)@4% AC	1	84	413.793	21.315	26.100
	3	25.6	194.825	11.680	92.391
	5	11.2	127.687	6.686	197.357
	7	5.2	54.2923	5.603	596.769
MnO_2_/Fe_3_O_4_ (1 : 1)@8% AC	1	106.8	515.113	27.679	20.966
	3	25.2	186.667	11.813	96.429
	5	12.2	128.808	7.864	195.639
	7	8	119.800	6.010	270.450
MnO_2_/Fe_3_O_4_ (1 : 1)@12% AC	1	68.8	322.500	18.347	33.488
	3	20.4	165.316	8.741	108.882
	5	8.6	86.246	5.836	292.186
	7	3.8	46.849	3.468	691.579

From the experimental findings, it was clear that the usual interaction between biomass-based activated carbon and transition metal-based oxides is critical to store charge in electrochemical energy-storage devices. Hence, the ternary composite MnO_2_/Fe_3_O_4_ (1 : 1)@8% AC-modified electrode demonstrated a superior charge-storage capability compared to the others. Its better charge-storage performance ability was also proved by visualizing its specific energy and specific power, as shown by Ragone plots (see Fig. 6k). The specific energy and specific power values of all the electrodes were calculated using eqn (3) and (4), respectively,^45^ and all the values at different current densities are mentioned in Table 4.3
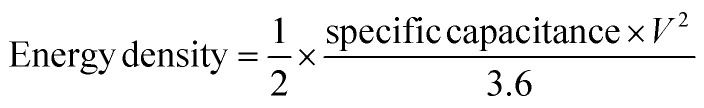
4
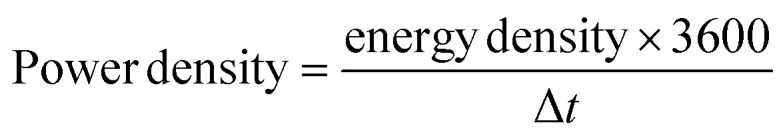
where *V* is the potential and Δ*t* is the discharge time. Therefore, the maximum energy density for MnO_2_/Fe_3_O_4_ (1 : 1)@8% AC was 27.679 Wh kg^−1^ at a current density of 1 A g^−1^ after 700 cycles. The cyclic stability of the optimized MnO_2_/Fe_3_O_4_ (1 : 1)@8% AC electrode was tested for 700 cycles, as shown in Fig. 6l. From the stability curve, it could be obtained that the maximum capacitive retention reached 81.83% after 700 cycles at a current density of 1 A g^−1^. All the determined values in these works are good and soundable due to low-cost and abunduant WH-based AC-modified MnO_2_/Fe_3_O_4_ as compared to transition metal oxide and sulfide-based electrode materials.^60–62^

#### 3.6.2. Energy-conversion analysis

**Fig. 7:**
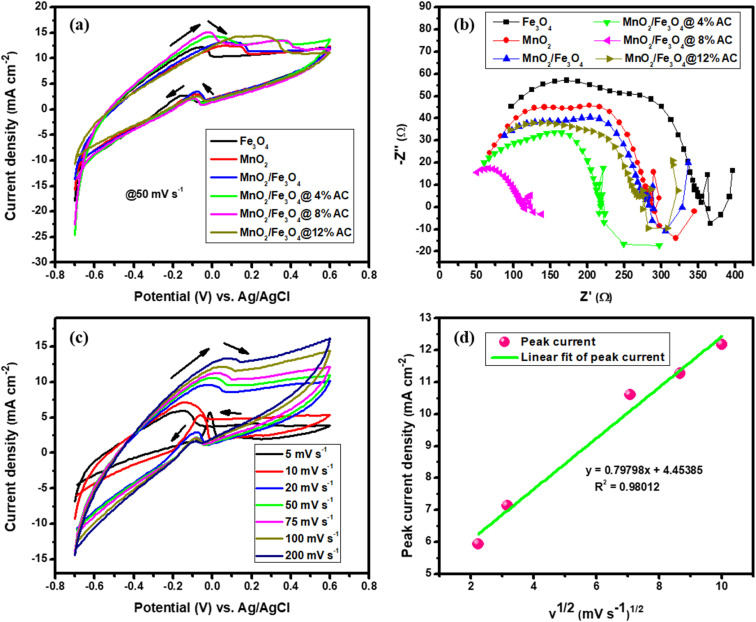
CV curves (a); Nyquist plots (b) of pure Fe_3_O_4_ (1 : 1) and MnO_2_ (1 : 1), and MnO_2_/Fe_3_O_4_ (1 : 1), MnO_2_/Fe_3_O_4_ (1 : 1)@4% AC, MnO_2_/Fe_3_O_4_ (1 : 1)@8% AC, and MnO_2_/Fe_3_O_4_ (1 : 1)@12% AC composites; CV curves of MnO_2_/Fe_3_O_4_ (1 : 1)@8% AC (c) at scan rates of 5, 10, 20, 50, 75, 100, and 200 mV s^−1^; and peak current density *versus* square root of the scan rate (d) in 1 M KOH containing 1 M C_2_H_5_OH.

The activities of the pure and composite catalysts for ethanol oxidation are shown in Fig. 7a. It is evident from this figure that the composite-based catalysts were better and had a higher current density than the pure metallic oxide-based catalysts due to the loading effect of the AC. In particular, this had a noticeable impact on the catalytic performance towards ethanol oxidation at a scan rate of 50 mV s^−1^ in the potential range between −0.7 and 0.7 V. The oxidation peak for the forward scan was due to the chemical adsorption of the freshly added ethanol and reached a maximum anodic peak current. Therefore, the reactive sites of all the catalysts decreased along with the anodic peak due to the blocking of the sites in the pure and composite-based catalysts, which caused a decrease in the adsorption of ethanol. In the forward scan, the electrons were released again and could react with the dissolved oxygen and in the reverse scan the reaction reduced in all catalytic surfaces based on the adopted reaction mechanism.^9,63^

5 Catalyst + C_2_H_5_OH + 3OH^−^ ⇌ Catalyst − CH_3_CO_ads_ + 3H_2_O + 3e^−^

6 Catalyst − CH_3_CO_ads_ + Catalyst −OH_ads_ + 6H_2_O + OH^−^ → 2Catalyst +CH_3_COO^−^ + H_2_O

where the catalyst was Fe_3_O_4_ (1 : 1), MnO_2_ (1 : 1), MnO_2_/Fe_3_O_4_ (1 : 1), or the MnO_2_/Fe_3_O_4_ (1 : 1)@4% AC, MnO_2_/Fe_3_O_4_ (1 : 1)@8% AC, and MnO_2_/Fe_3_O_4_ (1 : 1)@12% AC composites. Among them, MnO_2_/Fe_3_O_4_ (1 : 1)@8% AC was found to be the optimal catalyst and showed the best and maximum anodic peak current due to having the lowest charge-transfer resistance with higher conductivity, as shown in Fig. 7b. Hence, the Nyquist plot for MnO_2_/Fe_3_O_4_ (1 : 1)@8% AC, especially in the higher frequency region, showed a small semicircle compared to the others, and the lowest charge-transfer resistance at the interface between the electrode and electrolyte, which facilitates fast electrolyte ion diffusion into the electrode. Such fast ion diffusion is an indicator of its best catalytic activity towards ethanol oxidation. The CV curves of the MnO_2_/Fe_3_O_4_ (1 : 1)@8% AC catalyst at different scan rates in 1 M KOH containing 1 M C_2_H_5_OH are shown in Fig. 7c. It could be seen that as the scan rates increased, there was an increase in the oxidation peak current followed by a positive shift in peak potential due to the ohmic drop generated at a high current density, which was indicative of the catalytic ability of the MnO_2_/Fe_3_O_4_ (1 : 1)@8% AC catalyst for the electro-oxidation of the ethanol oxidation intermediates, such as acetaldehyde and acetic acid.^3^ Furthermore, a linear relationship was observed between the peak current and square root of the scan rates, with a regression coefficient of 0.98012 (see Fig. 7d), which was indicative of a diffusion-controlled process.^64^

**Fig. 8:**
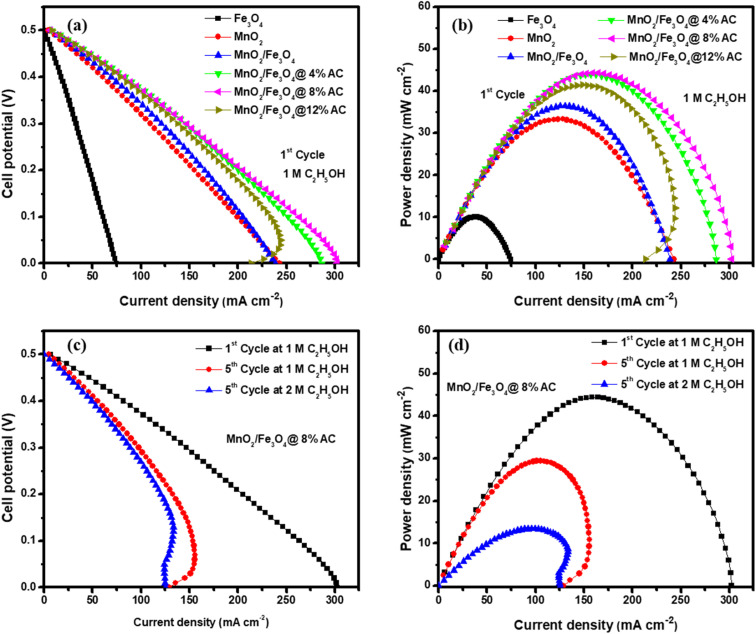
Fuel cell polarization curves (a) and power density curves (b) for Fe_3_O_4_ (1 : 1), MnO_2_ (1 : 1), MnO_2_/Fe_3_O_4_ (1 : 1), MnO_2_/Fe_3_O_4_ (1 : 1)@4% AC, MnO_2_/Fe_3_O_4_ (1 : 1)@8% AC, and MnO_2_/Fe_3_O_4_ (1 : 1)@12% AC as anode catalysts in 1 M KOH electrolyte containing 1 M C_2_H_5_OH; polarization curves (c) and power density curves (d) for MnO_2_/Fe_3_O_4_ (1 : 1)@8% AC anode catalysts in 1 M KOH containing 1 M C_2_H_5_OH and 2 M C_2_H_5_OH.

The steady-state polarization and power density curves of the different catalysts for ethanol electro-oxidation at room temperature and at 1 atm atmospheric pressure are shown in Fig. 8. Fig. 8a shows the polarization curves of all the catalysts in the first cycle with a constant ethanol concentration. The cell voltage dropped as the current density of the cell increased gradually, and from the shape of the curve, it could be observed that the activation polarization, ohmic polarization, and concentration polarization found at low, linear decreasing (middle curve), and high current densities, respectively, were best for the composite-modified catalysts. Due to the fast charge transfer kinetic properties found on the electrode. Due to the synergic surface modification between the metallic oxides and activated carbon, the concentration resistance was too low using MnO_2_/Fe_3_O_4_ (1 : 1)@4% AC, MnO_2_/Fe_3_O_4_ (1 : 1)@8% AC, and MnO_2_/Fe_3_O_4_ (1 : 1)@12% AC as anode catalysts. Hence, at the first cycle, a maximum power density of 44.41 mW cm^−2^ was obtained at a current density of 158.60 mA cm^−2^, as shown in Fig. 8b. As the number of cycles increased at a constant ethanol concentration, the catalytic performance of the best MnO_2_/Fe_3_O_4_ (1 : 1)@8% AC decreased to a power density of 29.46 mW cm^−2^ at a current density of 105.20 mA cm^−2^, as shown in Fig. 8c and d in the polarization curves. This might be due to a combination of different factors, including catalyst poisoning by the reaction intermediates, gradual degradation of the electrode materials, mass-transfer limitations, and a potential build-up of reaction byproducts, all of which may hinder the efficient oxidation of ethanol at the anode, ultimately reducing the current generation and power output.^10,65,66^ On the other hand, in the same cycles but with varying the ethanol concentration, the fuel cell with the MnO_2_/Fe_3_O_4_ (1 : 1)@8% AC catalyst showed the lowest power density of 13.44 mW cm^−2^ at a current density of 96.00 mA cm^−2^ due to limitations in mass transfer. It was evidenced that as the ethanol concentration increased, the rate of ethanol diffusion to the anode catalyst for electro-oxidation also increased, leading to a saturation effect, whereby the catalyst sites became overwhelmed and could no longer efficiently oxidize all the available ethanol molecules.^11,67^ A general comparison of the ethanol electro-oxidation performance of the MnO_2_/Fe_3_O_4_ (1 : 1)@8% AC catalyst in this work with other metal hybrid catalysts in other works is shown in Table 5.

**Table 5:** Comparison of the polarization and power density activities for ethanol electro-oxidation of the system reported in this study with those reported in other works

Catalyst	Power density (mW cm^−2^)	References
Pd-Ni_2_Pt/C-30%	90	3
Pd Cu TPPCu	0.4	10
Pt-Sn/C	30	66
Pt-Re-Sn/C	22.37	11
Pt_2_Sn_1_/C	60	68
PtSnO_2_	1.2	12
Pt-Ru/f-MWCNT	9.52	13
Pt/Rb-C/Sb	23.27	14
MnO_2_/Fe_3_O_4_ (1 : 1)@8% AC	44.41	This study

## 4. Conclusions

Sustainable energy-storage and -conversion devices using low-cost and environmentally friendly transition metal oxides, such as MnO_2_ and Fe_3_O_4_, have emerged as an efficient strategy for electrochemical systems. The low cost, large potential window, environmental friendliness, natural availability with high theoretical capacity of MnO_2_, and the presence of different valence states in Fe_3_O_4_ nanoparticles make them among the most attractive alternative electrode materials. The low electrical conductivity performance of MnO_2_ and Fe_3_O_4_ and short life cycle performance in MnO_2_/Fe_3_O_4_ binary composites, but having it with good energy density by storing the charge of the redox reaction; modification of Fe_3_O_4_/MnO_2_ with good conductive, low-cost, and long cycle life biomass-based AC has been paid considerable attention for energy storage applications. The hierarchical porous structure of AC incorporated with MnO_2_/Fe_3_O_4_ (1 : 1) to form ternary composites enhances the electrocatalytic application of these electrode materials in DAFCs (*i.e.*, ethanol electro-oxidation in this study). Hence, to overcome the charge-storage capacity and ethanol oxidation performance limitations mentioned above, composites of MnO_2_/Fe_3_O_4_ (1 : 1) with AC (4%, 8%, 12% wt) to form ternary composites were prepared using WH leaf extract as a reducing agent for hybridizing with carbonized AC. For comparison, pure MnO_2_ (1 : 1) and Fe_3_O_4_ (1 : 1) nanoparticles and MnO_2_/Fe_3_O_4_ (1 : 1) binary composite were synthesized as well. Thermal stability structural analysis was performed and showed that the intensity of the peaks related to the MnO_2_/Fe_3_O_4_ (1 : 1) phase changed as the AC content was increased in the composite samples. This was due to the heterogeneity of the different elements (Mn and Fe) and two non-metals (C and O) from the formed ternary composites, as evidenced by EDX. Such elemental distribution could be the reason for change in surface morphology with diverse porous structures, as illustrated by the BET results. Such surface and structural modifications enhanced the catalytic and ionic mobility by reducing the charge-transfer resistance, as evidenced by CV and EIS characterizations. Hence, clear redox peaks were observed using CV, which were due to the faradaic reactions, indicating the dominant pseudocapacitive behavior of the supercapacitors, with a specific capacitance and energy density of 515.113 F g^−1^ and 27.679 W h kg^−1^, respectively, at 1 A g^−1^. This performance, which reached a maximum of 81.83% capacitive retention after 700 cycles, was greatest when using the MnO_2_/Fe_3_O_4_ (1 : 1)@8% AC electrode. Using the same electrode, a maximum power density of 44.41 mW cm^−2^ was exhibited during ethanol oxidation in 1 M KOH electrolyte. Therefore, it was concluded that the MnO_2_/Fe_3_O_4_ (1 : 1)@8% AC electrode was the best performing electrode material for both charge-storage and energy-conversion applications.

## Data availability

All the data in this study are found in the main manuscript.

## Author contributions

Yilkal Dessie: Writing – review & editing, writing – original draft, software, resources, project administration, methodology, investigation, formal analysis, data curation, conceptualization. Eneyew Tilahun Bekele: conceptualization, methodology, data curation, validation, writing – review & editing, supervision. Bedasa Abdisa Gonfa: conceptualization, data curation, writing – review & editing, supervision. C. R. Ravikumar: data curation, visualization, resources. Syed Khasim: data curation, visualization, resources. Getahun Abraham: methodology, data curation, writing – review & editing.

## Conflicts of interest

There are no conflicts of interest to declare for all the authors.

## Supplementary Material

RA-015-D5RA02075A-s001
