# Supplementary material for: Electrochemical performances of MnO2/Fe3O4/activated carbon ternary composites for supercapacitor and direct ethanol fuel cell catalyst application
Source: RSC Adv. 2025 May 16;15(21):16493–509. doi: 10.1039/d5ra02075a (PMC12083547; doi:10.1039/d5ra02075a)
Supplement: RA-015-D5RA02075A-s001 [file RA-015-D5RA02075A-s001.pdf]

This journal is © The Royal Society of Chemistry 2025

Supplementary materials

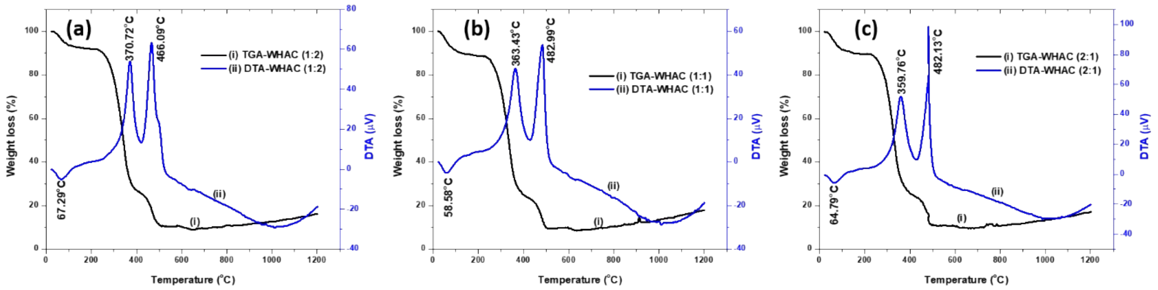

Fig. S1. TGA-DTA thermograms of (a) WHAC (1:2), (b) WHAC (1:1), and (c) WHAC (2:1).

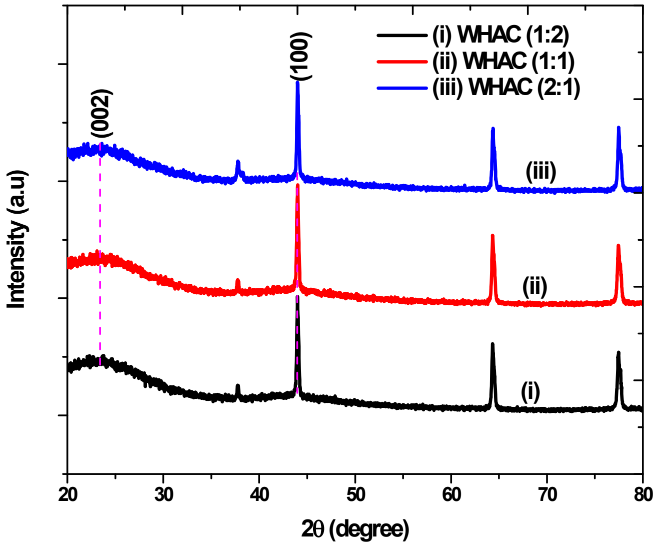

Fig. S2. XRD patterns of (i) WHAC (1:2), (ii) WHAC (1:1), and (iii) WHAC (2:1).

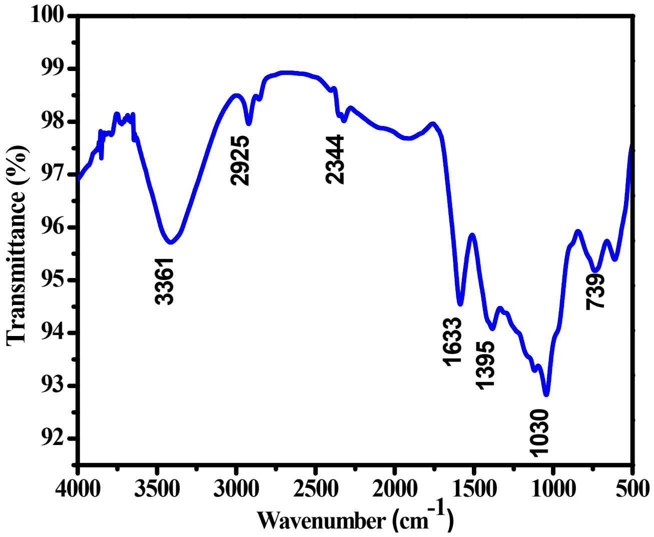

Fig. S3. XRD patterns of (i) WHAC (1:2), (ii) WHAC (1:1), and (iii) WHAC (2:1).

Table S1. Summary of average crystalline size for all prepared samples.

**Sample**	**Average crystalline size (nm)**
WHAC(1:2)	21.40
WHAC (1:1)	21.64
WHAC (2:1)	21.11
MnO_2 (1:3)	7.00
MnO_2 (1:1)	5.14
MnO_2 (3:1)	5.77
Fe_3O_4 (1:10)	8.10
Fe_3O_4 (1:1)	12.6
Fe_3O_4 (10:1)	9.80
MnO_2/ Fe_3O_4 (1:2)	9.10
MnO_2/ Fe_3O_4 (1:1)	8.30
MnO_2/ Fe_3O_4 (2:1)	13.50
MnO_2/ Fe_3O_4 @ 4% AC	10.40
MnO_2/ Fe_3O_4 @ 8% AC	7.65
MnO_2/ Fe_3O_4 @ 12% AC	13.10
